# Recent advancement of bioinspired nanomaterials and their applications: A review

**DOI:** 10.3389/fbioe.2022.952523

**Published:** 2022-09-08

**Authors:** Gang Wu, Xiaodan Hui, Linhui Hu, Yunpeng Bai, Abdul Rahaman, Xing-Fen Yang, Chunbo Chen

**Affiliations:** ^1^ Department of Critical Care Medicine, Maoming People’s Hospital, Maoming, Guangdong Province, China; ^2^ Center of Scientific Research, Maoming People’s Hospital, Maoming, Guangdong Province, Guangdong Province, China; ^3^ School of Food Science and Engineering, South China University of Technology, Guangzhou, China; ^4^ School of Public Health, Southern Medical University, Guangzhou, China; ^5^ Department of Intensive Care Unit of Cardiovascular Surgery, Guangdong Cardiovascular Institute, Guangdong Provincial People’s Hospital, Guangdong Academy of Medical Sciences, Guangzhou, China; ^6^ Department of Critical Care Medicine, Guangdong Provincial People’s Hospital, Guangdong Academy of Medical Sciences, Guangzhou, China; ^7^ The Second School of Clinical Medicine, Southern Medical University, Guangzhou, China

**Keywords:** bioinspired nanoparticles, biomimetic nanoparticles, natural nanoparticles, quantum dots, camouflage nanoparticles

## Abstract

With the advancement in the field of nanotechnology, different approaches for the synthesis of nanomaterials have been formulated, among which the bioinspired or biomimetic nanoplatforms have been utilized for different biomedical applications. In this context, bioinspired or biomimetic nanoparticles (NPs) have been synthesized in which the inspiration for synthesis is taken from nature or its components. Innovations in bioengineering tools and bio-conjugation chemistry have enabled scientists to develop novel types of such nanoplatforms. They have several advantages over normal synthesis protocols. In this review, we 1) summarized nanomaterial types and their advancements in bioinspired nanotechnology therapies; 2) discussed the major types, novel preparation methods, and synthesis progress of NPs in current biomedical fields; 3) gave a brief account of the need for synthesizing NPs via a bioinspired route rather than their common route; 4) highlighted the updated information on the biomimetic synthesis of different types of NPs; and 5) provided future perspectives in the synthesis of novel NPs for their potential applications in biomedical sciences.

## Introduction

In recent years, the demand for the use of nanotechnology in treating diseases such as cancer has been increasing, owing to the vast striking properties of nanomaterials that allow scientists to modify them to suit their needs. The size of therapeutically used nanoparticles (NPs) is lower than 100 nm with a specific surface area to volume ratio, which makes them a remarkable carrier for drugs as the positively charged particle is more compatible and attracted to negatively charged membranes of cells, which contributes to its higher cellular uptake. Nanomaterials tend to enhance the permeation and retention (EPR) effect, leading to good contact with cells and their compartments. Size and surface properties can be easily adjusted in these nanomaterials due to which different types of structures can be drawn, such as particles, fibers, and rods (Kim et al., 2010). NPs deliver drugs either passively or actively. In this respect, various nanomaterials have been employed to synthesize different types of NPs to apply them in the field of nanomedicine. However, due to stringent preparation methods and the use of harsh chemicals, the applicability of NPs is sometimes questionable, which leads to bioinspired methods coming into this picture. The most common members of bioinspired nanoparticles include solid lipid nanoparticles, dendrimers, aptamers, protein NPs, and viral NPs (Sivarajakumar et al., 2018) (Madamsetty et al., 2019). NPs have greatly improved the therapeutic action of many drugs and diagnostic value of various diseases due to its small size, large surface area–to-volume ratio, enhanced drug loading, easy synthetic routes, increased drug release timings, easy penetration abilities, and finally easy retention in the affected tissues.

**FIGURE 1:**
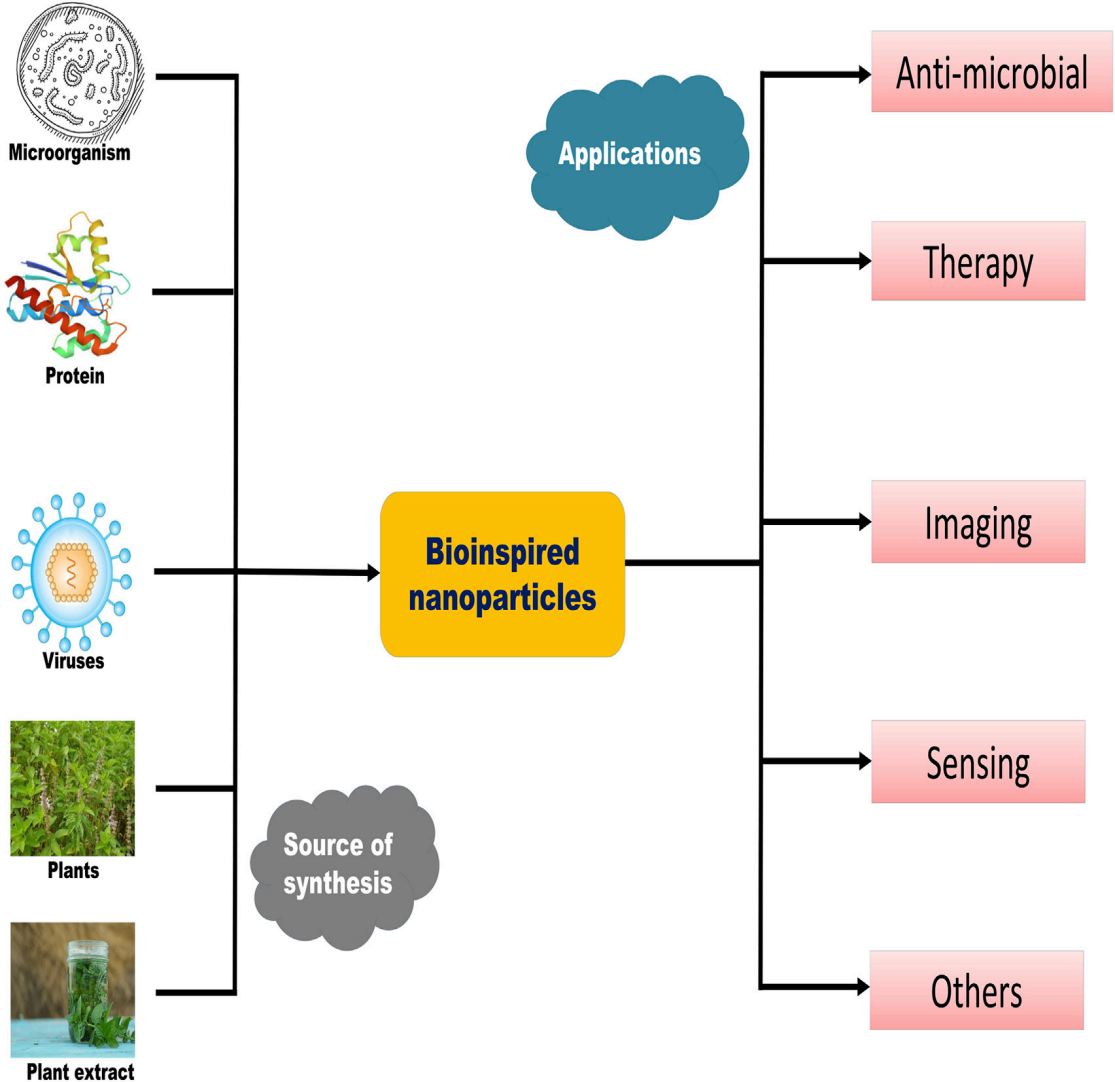
Illustrates different plasma membrane coatings on different nanoparticles for cancer immunotherapy. The plasma membranes of different types of cells like RBC, WBC, and cancer cells were extruded and coated with different types of nanoparticles (liposomes, dendrimers, carbon dots, polymeric nanoparticles, metallic nanoparticles, etc.) to form biomimetic nanoparticles which evoke immune responses (T cells, B cells, dendritic cells) to participate in the process of cancer immunotherapy.

Many diseases are caused by irregularities in the body at the molecular level or on a nanoscale, such as misfolding of important proteins, mutations in single nucleotide bases, and eventually infections induced by some pathogens (Kim et al., 2010). NPs have been given more attention due to the fact that they have the same size scale as biological molecules or components (Chan, 2017). Due to their tunable properties such as shape, size, morphology, surface charge, and surface elements, NPs can be used as therapeutic agents in the field of nanomedicine (Kim et al., 2010). Having some inspiration from biological aspects and the field of materials technology, bioinspired nanomaterials and their components, such as bioinspired nanoparticles and bioinspired nanovesicles, have received much more attention for two decades (Gaharwar et al., 2014). These materials, after mimicking nature, changed into novel generations of materials such as bacterial-inspired, mammalian cell–inspired, and virus-inspired nanosystems. Common nanosystems formed include lipid-based systems, vesicle-based nanosystems (exosomes), polysaccharide-based systems, and metallic nanosystems. The terms “biomimetic” and “bioinspired” are used interchangeably, with the same meaning but very little difference. The former states directly mimic techniques or processes that are present in nature, while the latter can be direct or indirect, with a wider range of uses and more flexibility. Figure 1 summarizes the synthetic sources and important applications of biomimetic nanomaterials.

**FIGURE 2:**
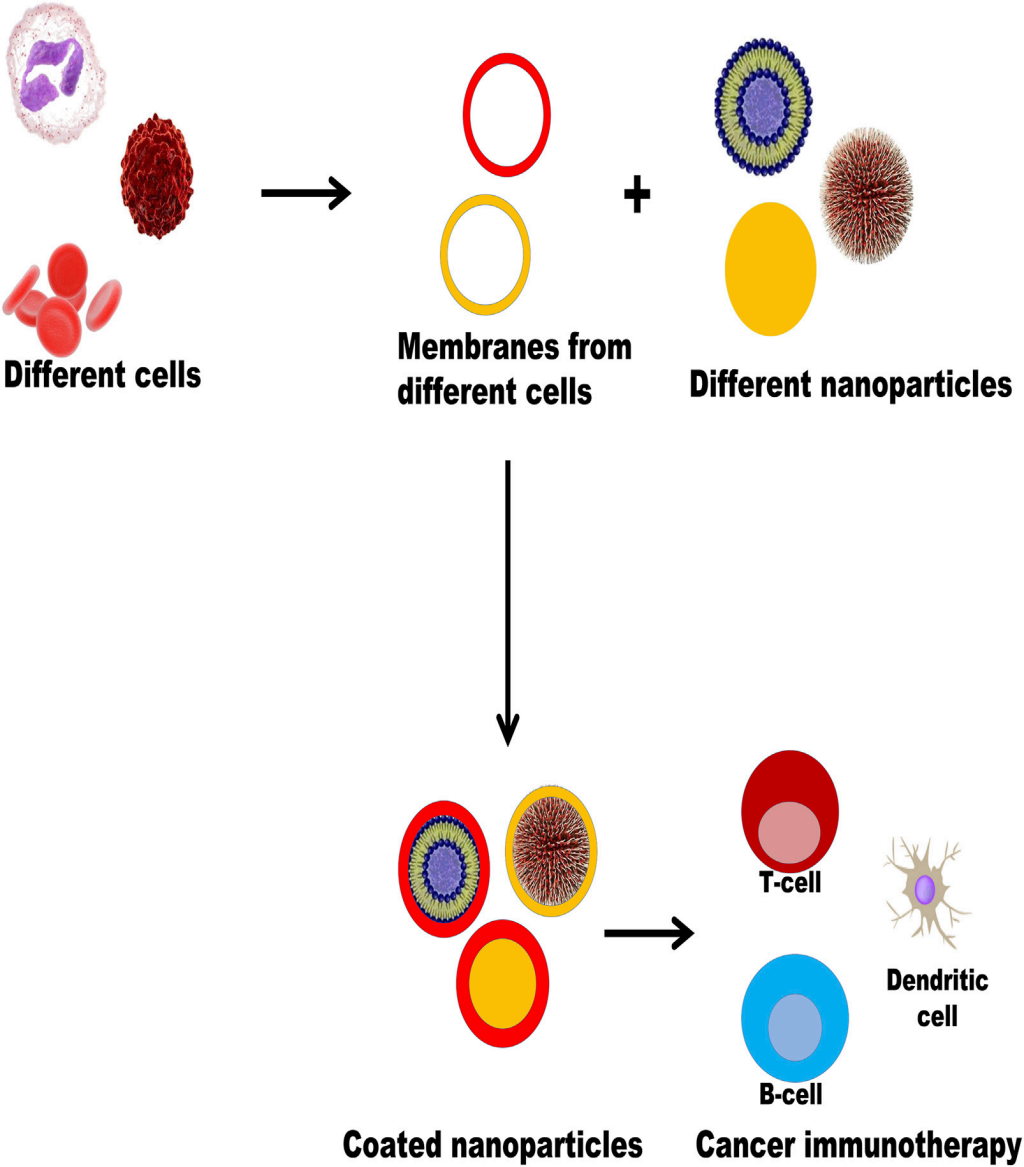
Illustrates different plasma membrane coating on different nanoparticles for cancer immunotherapy.

Currently, different types of cells such as red blood cells, white blood cells, cancer cells, and platelets are extruded from the plasma membrane and coated with different types of NPs by different types of technologies, such as liposomes, metallic NPs, dendrimers, quantum dots, and polymeric NPs, to form biomimetic NPs. These have been used to evoke immune responses involved in cancer immunotherapy. Figure 2 illustrates different plasma membrane coatings on different NPs for cancer immunotherapy.

In this article, various types of nanomaterials and their advancements in bioinspired nanotechnology therapy were critically reviewed. The major types, novel preparation methods, and synthesis progress of NPs in the current biomedical field were also discussed. Furthermore, we have given a brief account of the need for synthesizing NPs *via* a bioinspired route rather than their common route. This review highlighted the updated information on the biomimetic synthesis of different types of NPs. Table 1. summarized the nanoparticles formed by bioinspired technology discussed in the review article.

**TABLE 1:** Summarizes the nanoparticles formed by bioinspired technology discussed in the review article.

Type of nanoparticle	Name of nanoparticle	Synthesis protocol	Application	References
Silver nanoparticle	Bioinspired AgNPs	*Elaeagnus umbellate* extract (EU) for reducing silver nitrate to silver nanoparticles.	Good killing effect against gram-positive and gram-negative strains of *Staphylococcus aureus* (*S. aureus*) and *Escherichia coli* (*E. coli*)	Ali et al., 2020
Gold nanoparticle	AuNPs coated with reduced graphene oxide.	*Syzygium cumini* seed extract (SCSE) to simultaneously reduce chloroauric acid and graphene oxide (GO).	Enhanced antibacterial and anticancerous activity on *Staphylococcus aureus* and *Bacillus subtilis* and human colorectal cancer cell line (HCT 116) and lung (A549) cancer cell line, respectively.	Kadiyala et al., 2018
Iron oxide nanoparticle	SPION-loaded silica nanocapsules	SPION-loaded silica nanocapsules based on a bimodal catalytic peptide surfactant stabilized nanoemulsion template method	Encapsulating iron oxide into silica nanocapsules simply signifies the drug delivery ability.	Wilson et al., 2021
Poly (lactic-co-glycolic acid) (PLGA) nanoparticles	Polymeric nanoparticles coated with programmed cancer cell membrane (BiNPs)	Stimulated cancer cells for over-expression of integrin expression on the outermost surface of cells and then coated polymeric nanoparticles membranes.	Enhanced circulation time, escape from immune system, and improved biocompatibility	Hu et al., 2011
Alginate NPs	Bioinspired alginate NPs	Microbubble-bursting method	Improvement in size and dispersity of formed NPs	Elsayed et al., 2015
Nanovesicle	PSMA-targeted “Hybrid” nanoparticles	Hybrid nanoparticles in which they loaded PSA cleavable prodrug doxorubicin (DOX-PSA).	Increased specificity, decreased tumor growth in *in vitro* and *in vivo* models compared to free forms	Ma et al., 2021
Nanovesicle	Polymeric nanovesicle (TPZ/AI-NV)	Used diblock copolymers for the synthesis of nanovesicle: one was chlorine e6 (Ce6)–modified PEG-polyserine, another one was PEG-poly (Ser-S-NI	Precise drug delivery and finally synergistic therapeutic effect was observed	Qian et al., 2017
Nanovesicle	Biomimetic nanovesicle coated by PD-1 receptors	First, they transfected HEK 293 T cells with plasmid to express PD-1 on the surface of cell membrane and secondly they synthesized nanovesicles by dialysis method using repeated extrusion process	Nanovesicles accumulate near the tumor regions and retard the tumor growth through the filtration of CD8^+^ T cells.	Zhang et al., 2018a
Exosomes	Melanoma (cancer of skin)-derived exosomes	Loaded with immunomodulatory CpG DNA displayed *antigens* on their surface	Better in eradicating tumor than either exosomes or DNA alone	Morishita et al., 2016
Exosomes	Withaferin A (WFA)–loaded exosomes targeted by conjugated it with folic acid	Bioinspired exosomes derived from bovine milk	Enhanced antitumor effect (74%) when compared to non-targeted exosomes (50%)	Munagala et al., 2016
Lipoproteins	Bioinspired lipoprotein particle bLP	Loaded both a photothermal agent (DiOC_18_ (Hu et al., 2010) (DiR) producing D-bLP NPs and an anticancer drug, namely, mertansine forming M-bLP	D-bLP remodeled the tumor stromal microenvironment (TSM) and M-bLP killed the tumor cells and inhibited tumor relapse and metastasis	Tan et al., 2019
Nanovehicle based particle	Bioinspired tumor-responsive theranostic nanovehicle (BTV)	A theranostic probe of photochlor (HPPH), a tumor-activated melittin pro-peptide (TM), and a ROS-responsive prodrug gemcitabine (RG) was loaded into a lipoprotein-based bioinspired nanovehicle	Drastic elimination of multiple immunosuppressive cells and enhanced infiltration of cytotoxic lymphocytes in tumor	Wang et al., 2021
Chitosan or calcium phosphate–based Nanoparticles	VitB12 was conjugated on chitosan or calcium phosphate–based NPs	Ionic gelation method	Oral absorption of insulin was highly enhanced	Ke et al., 2015 *,* Verma et al., 2016
Polycaprolactone nanoparticles	Polyhydroxybutyrate/poly-3-caprolactone (PHB/PCL) mats	Process of electrospinning	Significant antimicrobial activity toward both the strains of bacteria (gram-positive/gram-negative), very good water holding capacity, hydrophilicity, and *in vitro* activity which clearly indicates its interaction and attachment	Avossa et al., 2021
Quantum dots	Fluorescent C-quantum dots	By the process known as hydrothermal approach using *Citrus limetta* juice.	Enhanced *in vitro* activity clearly indicates its anti-adhesion and anti–biofilm production ability of *Candida albicans*	Shaikh et al., 2019

## Bioinspired metallic nanoparticles

### Bioinspired silver nanoparticles (AgNPs)

Ali et al. (2020) used different fractions of *Elaeagnus umbellate* extract (EU) to reduce silver nitrate to silver NPs, ultimately synthesizing AgNPs. The formed NPs were morphologically controlled, and their shape/size-dependent application was evaluated. Furthermore, the shape, size, and bactericidal activity of these NPs were evaluated, and their mechanism of action was studied via atomic force microscopy (AFM) and scanning electron microscopy (SEM). They found that the NPs were around 40 nm in size and were monodisperse and non-toxic in nature. In addition, they had a good killing effect against gram-positive and gram-negative strains of *Staphylococcus aureus* (*S. aureus*) and *Escherichia coli* (*E. coli*). There was an electrostatic interaction between the bacterial cell wall and NPs. The results showed that the cell surface accumulation of NPs in *E. coli* was faster and more obvious than that in *S. aureus*, which was probably related to the different composition of cell walls of two different bacterial strains. NPs penetrated into bacteria and interacted with sulfhydryl groups to denature proteins, ultimately affecting enzyme activity.

### Bioinspired gold nanoparticle

Graphene has become one of the most developed nanomaterials and has shown great scientific value for future applications of nanotechnology (Mao et al., 2013). It may be considered one of the best biocompatible nanoplatforms due to its applications in antibacterial (Akhavan and Ghaderi, 2010; Hu et al., 2010; Ma et al., 2011), antiviral materials (Akhavan et al., 2012), cancer-targeting (Yang et al., 2010), drug delivery (Zhang et al., 2010), and photothermal therapy (Yang et al., 2012). A team of scientists synthesized gold nanoparticles (AuNPs) coated with reduced graphene oxide. They used *Syzygium cumini* seed extract to reduce both chloroauric acid and graphene oxide (GO). Meticulously, biophysical techniques such as UV-Vis spectroscopy (UV-Vis), dynamic light scattering (DLS), and Fourier transform infrared spectroscopy (FTIR) were employed to characterize its physicochemical properties. The results showed AuNPs were successfully synthesized and coated with graphene oxide. The antibacterial and anticancerous activities were performed on the strain of gram-negative bacteria, *E. coli*, and on the strains of gram-positive bacteria, *S. aureus* and *Bacillus subtilis*, and on the human colorectal cancer cell line (HCT 116) and lung (A549) cancer cell line, respectively. The cytotoxicity and antibacterial toxicological assays revealed that the synthesized nanocomposite showed significant anticancer activity against the A549 cell line and gram-negative bacterial strain *E. coli* compared to the rest of the strains (Kadiyala et al., 2018).

### Bioinspired iron oxide nanoparticles

Superparamagnetic iron oxide NPs (SPIONs) have been extensively used owing to their unique properties. However, their use in the biomedical field is hampered by the fact that these NPs are more toxic, less magnetic, and expensive due to the harsh chemical reagents used for the synthesis of these NPs. Russell J. Wilson bioengineered SPION-loaded silica nanocapsules based on a bimodal catalytic peptide surfactant stabilized nanoemulsion template. SPIONs were preloaded into the oil phase of nanoemulsions, and the surface property of the peptide and its electrostatic repulsion resulted in the stability of nanoemulsions. Catalytic peptides lead to biosilification and promote the formation of silica shell nanocapsules containing iron oxide. In conclusion, the encapsulating of iron oxide into silica nanocapsules simply signifies the drug delivery capabilities of these formed nanoplatforms (Wilson et al., 2021).

### Bioinspired green synthesis of metallic nanoparticles

Inspired by nature, there is a growing opportunity for bioinspired synthesis of metallic nanoparticles due to its ease and low cost of biosynthesis, and they can be easily modified by many proteins, lipids, carbohydrates, and antibodies to enhance their biological effects (Gurunathan, 2019). This is attributed to the toxic effects and high energy inputs provided by the use of harsh chemicals and stabilizers in the synthesis and processes of these metallic NPs. From the point of view of industrial preparation, it is necessary to ensure that the NPs are well dispersed and their size is controlled. Various attempts have been made to utilize many food by-products such as orange peels (Castro et al., 2013) and banana peels (Ibrahim, 2015). In addition to their use in synthesis as reducing agents, they can be easily recovered into some useful products through these processes. Inspired by these facts, a group of workers synthesized silver (Ag), gold (Au), and platinum (Pt), using an aqueous extract of the rind of the fruit *Garcinia mangostana*, used against inflammation, cholera, and diarrhea (Pedraza-Chaverri et al., 2008), to check for their antimicrobial activity with or without their attachment to several classes of antibiotics. The results showed that AgNPs had a better antimicrobial effect than AuNPs and PtNPs against gram-negative strains of bacteria, and all groups of NPs showed synergistic activity with different classes of antibiotics, indicating a certain correlation between antibiotics and NPs. Additionally, *Bacillus spp.*, previously found to be resistant to streptomycin, was now susceptible to the combination of AuNPs and antibiotics. Collectively, metallic NPs increased the susceptibility of bacterial strains to antibiotics (Nishanthi et al., 2019).

## Polymeric bioinspired nanoparticles

### Poly (lactic-co-glycolic acid) nanoparticles

Organ-specific drug targeting remains a challenging task in the fields of drug delivery and nanomedicine. A team of scientists has synthesized programmable bioinspired NPs (P-BiNPs) that can deliver cargo to homotypic cancer cells while targeting bone localization in animal models. First of all, they stimulated cancer cells to over-express integrin on the outermost surface of cells. They then coated the polymeric NPs with these programmed cancer cell membranes, which were absorbed by prostate cancer cells to improve the therapeutic ability of drugs, enhance their imaging quality, and ultimately reduce side effects. Coating these NPs in biologically inspired nanomaterials enhanced their circulation time, escaped from the immune system, and improved biocompatibility (Hu et al., 2011).

### Alginate nanoparticles

In the field of nanotechnology, natural and synthetic NPs have been extensively synthesized and characterized for drug delivery. It comprises poly (D,L-lactide), poly (D,L-glycolide), poly (lactic acid), poly (lactide-co-glycolide) acid, alginate, chitosan, gelatin, and collagen (Soppimath et al., 2001). Alginate NPs are an important class of polymeric drug delivery carriers that enhance bioavailability and finally the efficacy of many drugs (Kulkarni Vishakha et al., 2012). Alginate, a natural sugar polysaccharide, is mucoadhesive in nature due to the cationic nature of the polymer so that it can adhere to the plasma membrane (Malafaya et al., 2007).

Synthesis schemes have been developed from time to time to obtain proper alginate NPs. However, these conventional methods lack proper regulation, require dispersion and size of NPs, and finally require the use of harsh organic solvents that might be toxic to the *in vivo* environment. Bubble bursting is a natural phenomenon occurring in the marine medium virtue, which forms nano-sized and micro-sized particles (Fitzgerald, 1991). It is mainly caused by wave breaking *via*, namely, bubble film disintegration and jetting (Spiel, 1998). Hence, alginate NPs have been synthesized using the microbubble-bursting method with a size range of 80–200 nm. A device, which is T junction microfluidic, was used by the researcher group to form microbubbles with varying sizes in the best possible controlled manner. The size produced was directly related to the viscosity of the alginate solution used in this process (Elsayed et al., 2015).

## Bioinspired nanovesicle

### Nanovesicles

Nanovesicles based on lipids have been widely used as important drug delivery carriers in the field of nanomedicine due to their good biocompatibility and sample preparation protocols. In nanovesicles, the outer layer is covered with two lipids and the inner part is composed of an aqueous cavity. The first and foremost characterized lipid nanovesicle was liposomes, and they have been used as drug delivery carriers to deliver genes and drugs (Grimaldi et al., 2016). Bioinspired nanovesicles include biomimetics and cell-derived nanovesicles, which form a new class of drug delivery carriers (Goh et al., 2017; Zhao et al., 2020). The construction of these vesicles involves extrusion of intact cells and then synthesis of nanoparticles with a coating of cell-derived membranes and fusing exosomes with lipid-derived particles (Ma et al., 2021). This ensures a high loading capability and mimics many natural particles so that it may not evoke any type of immune response. Finally, they are also highly biocompatible, finding application in drug delivery, immunotherapy, tumor targeting, and gene delivery (Ilahibaks et al., 2019; Park et al., 2019).

Ma et al. (2021) simultaneously targeted two specific components of prostate cancer (PC). In PC, prostate-specific membrane antigen (PSMA) and prostate-specific antigen (PSA) are found to be highly upregulated in advanced stages of PC, and there was no evidence that they are both targeted. Hence, they designed PSMA-targeted “Hybrid” NPs and loaded them with the PSA cleavable prodrug, doxorubicin (DOX-PSA). The specificity of *in vitro* and *in vivo* models increased and tumor growth decreased compared to free forms and untargeted PSA hybridization, indicating an enhanced efficacy of the formed nanovesicle loaded with the prodrugs.

Inspired by anaerobic bacteria and their metabolism under hypoxia, Qian et al. (2017) synthesized the nanovesicle system. Under hypoxia, external light irradiation delivered the material to the tumor microenvironment, resulting in a reaction. They used diblock copolymers for the synthesis of nanovesicles: one was chlorine e6 (Ce6)–modified PEG-polyserine, and the other was PEG-poly (Ser-S-NI). When the light irradiates the photosensitizer Ce6, oxygen is converted to singlet oxygen, which is further consumed by oxidizing the thioether on PEGpoly (Ser-S-NI) to a hydrophilic oxidized state, resulting in an anoxic atmosphere. This low oxygen concentration atmosphere could bring out the bioreduction of NI pendants into hydrophilic units and eventually the disassociation of the nanovesicles. Additionally, by encapsulating the hypoxia-activated prodrug tirapazamine into the cavity of nanovesicles, automated, precise drug delivery and finally synergistic therapeutic effect between the two main processes, namely, photodynamic therapy and chemotherapy could thus be achieved.

Nanovesicles derived from cell membranes can be directly used for biomimetic nanomedicines. Taking advantage of genetic engineering technology and nanotechnology, Zhang and co-workers recently developed a biomimetic nanovesicle that represents the PD-1 receptor on its surface for cancer immunotherapy. First, HEK 293 T cells were transfected with a plasmid to express PD-1 on the surface of the cell membrane, and then nanovesicles were synthesized by the dialysis method using a repeated extrusion process. The blockade of PD-1 or PD-L1 is an emerging trend in cancer immunotherapy as it suppresses the host antitumor immune response. Data from their experiments revealed that vesicles carrying PD-L1 bind to PD-L1 receptors on cancer cell membranes. In addition, *in vivo* studies have shown that these nanovesicles accumulate near tumor regions and retard tumor growth through filtration of CD8^+^ T cells. This system was boosted by the use of drugs such as 1-methyl-tryptophan, which was proved to be an effective inhibitor of the immunosuppressive enzyme indoleamine 2, 3-dioxygenase (IDO) (Pardoll, 2012). By encapsulating it in the core of a nanovesicle, they have increased the efficacy of formed particles by blocking two important pathways (Zhang et al., 2018a).

### Extracellular vesicles

Extracellular vesicles are heterogeneous entities released by cells and play a key role in cell-to-cell communications. Exosomes are the smallest of all kinds of extracellular vesicles, with a size ranging from 50 to 150 nm (Stremersch et al., 2016). They are used as drug delivery agents as they can be moved from one location to another and in some cases, as a diagnostic marker. However, it is sometimes difficult to use it as a sole drug delivery agent due to challenges faced by many scientists, such as low loading capacity and obtaining a lower amount of exosomes in normal conditions. Hence, bioinspired exosomes come into play as an alternative to naturally derived exosomes, and it proved to be an effective therapy against many issues, as mentioned previously (Lu and Huang, 2020).

### Exosomes

It has been reported that exosomes from different types of cells, such as those derived from immune cells and mesenchymal stem cells (MSCs) in particular conditions, possess different therapeutic responses (Buschow et al., 2010; Sun et al., 2018). In this regard, exosomes derived from B cells present a major histocompatibility complex on their heads, so the induction of T-cell responses (Clayton et al., 2003) indicates that exosomes could be used as an immunomodulatory agent, which was proved by dendritic cell (DC)–derived exosomes added with tumor antigens, evoking immune responses and inhibiting the survival of established tumors (Zitvogel et al., 1998). Taking these effects as an immunomodulatory and immunotherapy agent, exosomes are used to load cargo, in addition to displaying antigens on their surface. For example, melanoma (cancer of the skin)-derived exosomes from murine models loaded with immunomodulatory CpG DNA displayed antigens on their surface which proved to be better in eradicating tumors than either exosomes or DNA alone (Morishita et al., 2016).

Bovine milk is used for the synthesis of cost-effectiveness and for the large-scale production of exosomes in a bioinspired manner. When withaferin A (WFA) was administered three times per week, enhanced antitumor activity was found in xenograft mice bearing A549 lung cancer. When exosomes were modified with the ligand folic acid (FA), the antitumor effect was enhanced (74%) when compared to non-targeted exosomes (50%) (Munagala et al., 2016). Other groups also successfully loaded different drugs, such as anthocyanidins and paclitaxel, for oral administration of milk-derived exosomes (Agrawal et al., 2017; Munagala et al., 2017).

## Miscellaneous

### Bioinspired lipoproteins

**FIGURE 3:**
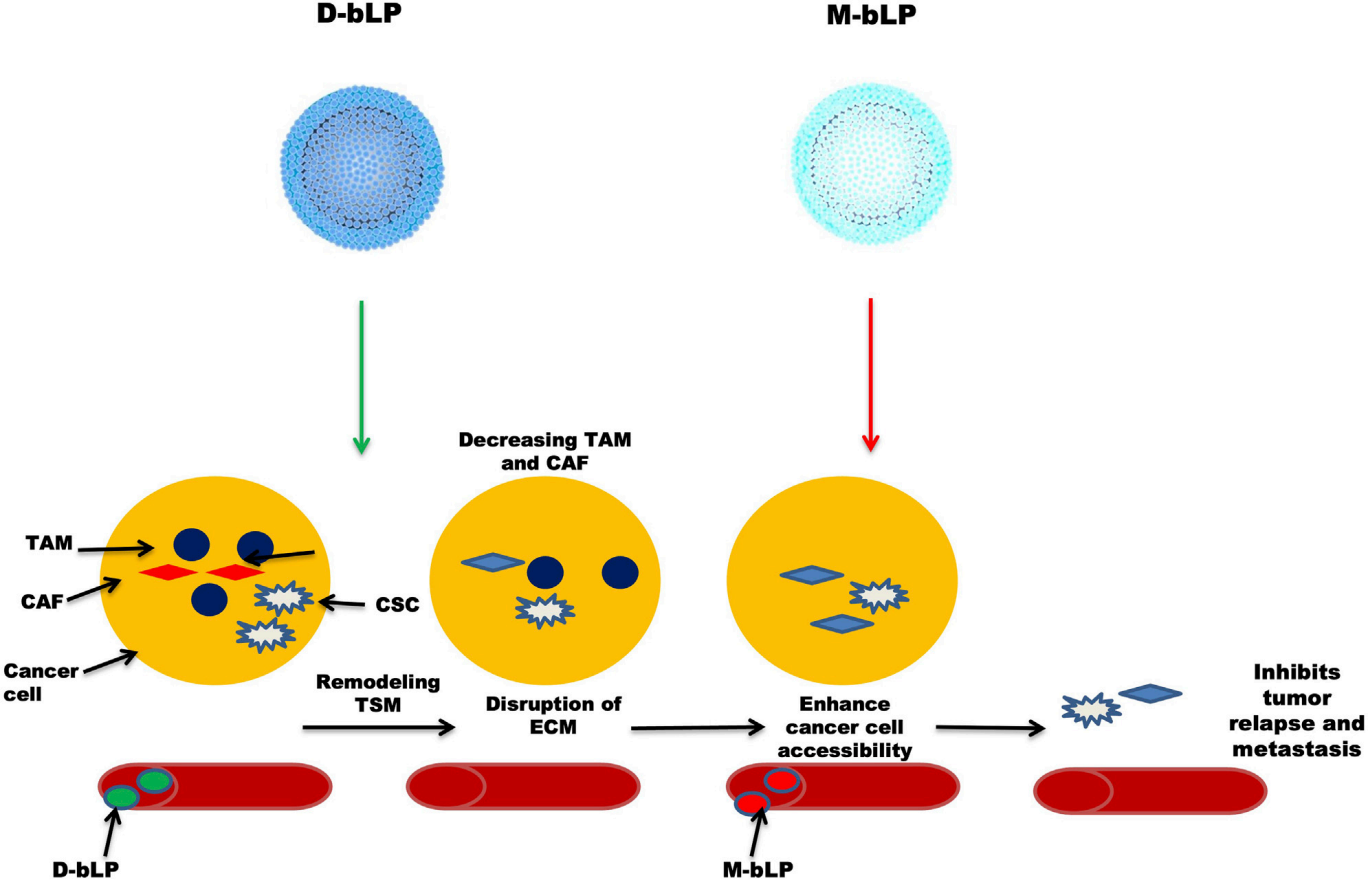
Illustrates D-bLP-mediates photothermal remodelling of tumor stroma which increases the accessibility of second wave of nanoparticles M-bLP near cancer cells.

In a cancer environment, several nanosystems are not effective in providing a therapeutic response due to the inability of many nanosystems to access cancer cells (Minchinton and Tannock, 2006; Dewhirst and Secomb, 2017). NPs passively accumulate near the tumor microenvironment, but only a few (around 5%) NPs actually reach the tumor site (Wilhelm et al., 2016; Dai et al., 2018). Researchers found that a large number of stromal cells, such as cancer-associated fibroblasts (CAF) and tumor-associated macrophages (TAM), are needed during the development of cancer (Kitamura et al., 2015; Kalluri, 2016). By forming an extracellular matrix (ECM), they play a pivotal role in shaping the morphology and the whole environment of tumor tissue. NPs are actually hijacked by ECM, preventing them from penetrating into tumor tissue and eventually lowering their efficacy in cancer therapeutics (Zhang et al., 2018b; Overchuk and Zheng, 2018). Lipoproteins, notably, the high-density lipoproteins (HDL) are endogenous nanoscale particles composed of a variety of proteins (e.g., apolipoprotein A1, Apo A1) and some lipids (e.g., phospholipids and cholesterol esters), making them an ideal platform for sustained delivery of many therapeutic agents and in biological imaging of tumor tissues. Tan et al. (2019) synthesized the bioinspired lipoprotein particle, bLP, which was loaded with a photothermal agent (DiOC_18_) (Hu et al., 2010) (DiR) to produce D-bLP NPs and the anti-cancer drug, namely, mertansine, to form M-bLP. These two were used one by one to ensure proper management of the disease. First, they administered D-bLP using a photothermic pulse in the infrared (IR) range to reshape the tumor stromal microenvironment (TSM), and then actively enhanced the second wave of M-bLP to kill the tumor cells and inhibit tumor relapse and metastasis, as done in two breast cancer models. Figure 3 illustrates D-bLP–mediated photothermal remodeling of tumor stroma which increases the accessibility of the second wave of M-bLP nanoparticles near cancer cells.

### Bioinspired theranostic tumor permeated nanovehicle

In addition to the challenges facing cancer treatment, there are several obstacles to effective therapy in the oncology field. This is probably due to the presence of certain types of immunosuppressive cells, namely, myeloid-derived suppressor cells (MDSCs), M2-like tumor-associated macrophages (TAMs), regulatory T cells (Tregs), and immature/tolerogenic dendritic cells (DCs), in the context of the tumor cell region that is considered immunosuppressive (Alizadeh and Larmonier, 2014; Shaked, 2019; Togashi et al., 2019). Cancer cells have also evolved a natural tendency to suppress the immune response of immunosuppressive cells that function as CD8^+^ T cells and natural killer (NK) cells in tumors through multiple mechanisms, thereby hampering the antitumor immunity (Binnewies et al., 2018; Shaked, 2019). Undoubtedly, there is a need to cope with this situation in which immunosuppression is relieved, enhancing the antitumor response of cancer cells.

Wang et al. (2021) synthesized a bioinspired tumor-responsive theranostic nanovehicle (BTV) with tumor-penetrating ability to cope with immunosuppression of cancer cells for effective anti-cancer therapy. In this nanovehicle, a theranostic probe of photochlor (HPPH), a tumor-activated melittin pro-peptide (TM), and an ROS-responsive prodrug gemcitabine (RG) were loaded into a lipoprotein-based bioinspired nanovehicle. The functions of different compounds are as follows: TM enhances tumor penetration and accumulation capacity and was enzymatically restored to active melittin at the specific sites, thereby increasing the activities of pharmacological drugs. RG (prodrug), as an active immunomodulator, was degraded into active gemcitabine. HPPH acted as a theranostic probe in BTN for systemic tumor tracking *in vivo* and generated singlet oxygen upon irradiation to enhance the overall antitumor activity of the formed nanovehicle. Remarkably, this combinational treatment significantly eliminated multiple immunosuppressive cells and enhanced the infiltration of cytotoxic lymphocytes in tumors, which is the essential key element in relieving tumor immunosuppression and also strikingly decreasing tumor growth. In a nutshell, this novel design provides a pathway to deliver a nanoplatform with striking immunosuppression-relieving capacity that could be used for effective anti-cancer therapies.

### Bioinspired VitB12-coated NPs

Receptor-mediated endocytosis, a process by which VitB12 is absorbed, has been reported by several groups (Seetharam, 1999). Hence, this vitamin is utilized for coating NPs to improve their oral bioavailability. Conjugation of this vitamin on insulin-encapsulated dextran NPs improved insulin availability (26.5%) in chemically induced diabetic rats (streptozotocin-induced) compared with control rats (10.3%) without any coating (Chalasani et al., 2007a; Chalasani et al., 2007b). Similar trends were observed when VitB12 was conjugated on chitosan or calcium phosphate–based NPs; oral insulin absorption was greatly enhanced (Ke et al., 2015; Verma et al., 2016), while poor oral availability of some drugs like cyclosporine A and scutellarin was also improved when NPs were coated with VitB12 (Francis et al., 2005; Wang et al., 2017).

### Bioinspired wound healing dressing mat

Wound treatment is challenging as some diseases, such as diabetes and cardiovascular diseases, make it more chronic (Qu et al., 2018). Wound dressing plays a key role in the healing process by mimicking ECM, adhesion, and eventually migrating to the wound, aiding in the process of healing and skin regeneration (Chhabra et al., 2016). Polycaprolactone (PCL) has been widely used in wound healing due to its good biocompatibility, biodegradability, and easy availability (Ravichandran et al., 2019). It can be blended with other polymers to improve its mechanical properties and tissue regeneration abilities (Sawadkar et al., 2020). In this regard, biodegradable and eco-friendly polyhydroxybutyrate/poly-3-caprolactone (PHB/PCL) mats were developed by electrospinning to imitate the extracellular matrix (ECM) and to provide structural and biochemical evidence for tissue regeneration. Inspired by the natural component melanin, which is highly exploited as a tool against microbial infection, the above-developed mats were modified by melanin–TiO_2_ nanostructures. These coated mats had significant antimicrobial activity toward both the strains of bacteria (gram-positive/gram-negative). They had good water holding capacity, hydrophilicity, as well as *in vitro* activity, indicating their interaction and attachment (Avossa et al., 2021).

### Bioinspired carbon dots

As a powerful carrier, quantum dots have been widely used in the biomedicine field due to their optical properties based on their size. The preparation methods are quite difficult, take a lot of time, and have low reproducibility; among these, the hydrothermal approach is one of the green chemistry approaches for producing fluorescent carbon quantum dots (C-dots) (Kasibabu et al., 2015) on a large scale by using waste material and natural resources (like fruit juice of orange, ginger, and sugarcane) as carbon starting materials. Additionally, watermelon peel, milk, lignin, sugarcane juice, coffee grounds, chicken eggs, food waste, banana, hair, ginger, onion waste, honey, bread, candle soot, chitosan, and gelatine have been utilized as carbon sources (Zhou et al., 2012; Li et al., 2014; Liu et al., 2014; Mehta et al., 2014; Wang and Zhou, 2014; Bandi et al., 2016). Considering these specialties of natural resources, Asiya F. Shaikh synthesized rapid, highly fluorescent C-dots using a hydrothermal approach, using *Citrus limetta* juice, commonly known as Mausambi in the Indian subcontinent. It contains a high amount of sugar as carbohydrate, which is the starting source of carbon for C-dot production. *In vitro* activity studies have shown that they have anti-adhesion and anti–biofilm production ability of *Candida albicans* grown on polystyrene surfaces. In a nutshell, this novel approach provides a new way to synthesize C-dots using natural sources of carbon (Shaikh et al., 2019).

## Viral nanoparticle

Nowadays, viruses are being employed in the synthesis of bioinspired/biomimetic nanoplatforms due to their unique properties. Viral nanomaterials can be synthesized using virus nanoparticles (VNPs) and virus-like particles (VLPs). The latter is being utilized for the synthesis of inorganic NPs and the delivery of drugs and bio-imaging agents (Allen et al., 2005; Liepold et al., 2007). A virus consists of a protein coat called the capsid, which is considered a smart material because of its monodispersity, symmetry, and polyvalency. Among the various types of viruses, the helical virus is a prefabricated scaffold with a unique structure and a high surface area–to-volume ratio that enables it to form various types of nanostructures (Narayanan and Han, 2017a). Plant virus capsids provide the best platform for the synthesis of novel nanomaterials that combine inorganic or organic moieties in a very specific and controllable way. In addition, the capsid proteins of spherical plant viruses are assembled into well-defined 3D structures called icosahedral three-dimensional architectures with structural symmetry. They can be employed for a wide range of biomedical applications with simple manipulations (Narayanan and Han, 2017b). Taking inspiration from this, TMV, a helical virus that causes tobacco mosaic virus disease in tobacco plants, is used to synthesize bioinspired nanomaterials. Due to its elongated hollow tube-like structure (4 nm in diameter), it can be easily turned into nanorods. The central hollow space is utilized for the synthesis of cobalt and nickel nanowires (3 nm in diameter). Tsukamoto and co-workers developed a novel formula to produce bimetallic Co-Pt and Fe-Pt alloy nanowires in the hollow channel of the TMV. The process of nucleation and the growth of Co-Pt and Fe-Pt nanowires were successfully examined and characterized in detail (Tsukamoto et al., 2007).

## Conclusion and future prospects

The present work describes the biosynthesis of NPs using approaches derived from natural sources or inspired by nature. Bioinspired NPs avoid several disadvantages of conventionally used protocols, including the use of harsh chemicals in their preparations. These NPs are less toxic, easy to prepare, and cost-effective. Novel types of bioinspired nanoplatforms have potential applications in the field of nanomedicine. Currently, they have various applications in the biomedicine field, such as cancer therapy, antimicrobial, immunotherapy, biosensing, and diagnosis. In the case of membrane coating, the membranes of cancer cells are coated with NPs so that the natural defense system is activated to produce cancer immunotherapies much like nano-vaccines. They may also benefit from increased blood circulation time, reticuloendothelial system escaping, and tumor-specific active targeting.

With the advancement of material science and nanotechnology, proper care should be taken to avoid any uncontrolled reactions leading to the formation of polydisperse and larger NPs, thus affecting their therapeutic effectiveness. Novel methods should be devised for the proper synthesis of bioinspired nanomaterials so as to formulate novel NPs with higher loading efficiency and better efficacy. In the future, antibodies, proteins, and peptides can be inserted into the plasma membrane of NPs to achieve targeted and improved therapeutic effects. Research should focus on translating the synthesis of NPs into clinical applications and mass production at a lower cost.

## Abbreviations

EPR, enhanced permeation and retention; AgNPs, silver nanoparticles; DLS, dynamic light scattering; SPION, superparamagnetic iron oxide NPs; PLGA, poly (lactic-co-glycolic acid; PSMA, prostate-specific membrane antigen; PSA, prostate-specific antigen; WFA, withaferin A; FA, folic acid; TAM, tumor-associated macrophages; CAF, cancer-associated fibroblast; ECM, extracellular matrix; HDL, high-density lipoproteins; Apo A1, apolipoprotein A1; TSM, tumor stromal microenvironment; BTV, bioinspired tumor-responsive theranostic nanovehicle; PHB/PCL, polyhydroxybutyrate/poly-3-caprolactone; C-dots, carbon quantum dots.
